# Crown and Bridge Work

**Published:** 1888-12

**Authors:** 


### Crown and Bridge Work.

A practical treatise on Artificial Crown and Bridge Work, by George Evans, with five hundred illustrations. This treatise, upon a very important and valuable branch of the dental art, comes at an opportune time; much interest has been excited in the profession in regard to this mode of inserting artificial teeth. A knowledge of the proper methods of constructing the work has not kept pace with the growing interest. This work by Dr. Evans will not only increase the interest in this kind of work but will enable those of ordinary skill and attainments to prepare and adapt the various styles of crown and bridge work. Much mischief has been done through the want of that which this treatise so well and clearly presents; viz.: a practical knowledge of the principals and mode of construction and application.

No one who wishes to keep up with the progress of the profession can afford to be without this work of Dr. Evans.

It is published by the S. S. White Manufacturing Co. and this suffices as to the style of the book.

